# Gene transfer and expression in human neutrophils. The phox homology domain of p47^*phox *^translocates to the plasma membrane but not to the membrane of mature phagosomes

**DOI:** 10.1186/1471-2172-7-28

**Published:** 2006-12-06

**Authors:** Jennifer L Johnson, Beverly A Ellis, Daniela B Munafo, Agnieszka A Brzezinska, Sergio D Catz

**Affiliations:** 1 Department of Molecular and Experimental Medicine, The Scripps Research Institute, La Jolla, USA

## Abstract

**Background:**

Neutrophils are non-dividing cells with poor survival after isolation. Consequently, exogenous gene expression in neutrophils is challenging. We report here the transfection of genes and expression of active proteins in human primary peripheral neutrophils using nucleofection.

**Results:**

Exogenous gene expression in human neutrophils was achieved 2 h post-transfection. We show that neutrophils transfected by nucleofection are functional cells, able to respond to soluble and particulate stimuli. They conserved the ability to undergo physiological processes including phagocytosis. Using this technique, we were able to show that the phox homology (PX) domain of p47^*phox *^localizes to the plasma membrane in human neutrophils. We also show that RhoB, but not the PX domain of p47^*phox*^, is translocated to the membrane of mature phagosomes.

**Conclusion:**

We demonstrated that cDNA transfer and expression of exogenous protein in human neutrophils is compatible with cell viability and is no longer a limitation for the study of protein function in human neutrophils.

## Background

Study of a gene product by expressing its constitutively active or dominant negative mutant in a cell is a powerful tool of investigation. However, neutrophils are non-dividing cells with poor survival after isolation. Consequently, exogenous gene expression in neutrophils is challenging. Researchers have partially overcome these difficulties by performing studies in well-developed cell-free systems [1], with permeabilized neutrophils [2] and with cell lines that undergo some neutrophil functions, such as HL-60 cells or B-lymphoblasts [3,4]. Others have used virus-based expression systems, but these systems require laborious cloning into specific vectors and the procedures for viral infection are time consuming and potentially hazardous [5]. Here we report the transfection and expression of genes into neutrophils by nucleofection. Using this technology, which delivers the vector directly into the nucleus [6], exogenous genes can be expressed in neutrophils in as short as 2 h after transfection, overcoming the difficulties associated with the short lifetime of these cells.

In innate immunity, neutrophils are crucial for the destruction of bacteria and fungi [7,8]. To combat bacterial infection, neutrophils must perform functions that include migration to the inflammatory site, phagocytosis of invading microorganisms, and generation of reactive oxygen species (ROS) that contribute to killing. The NADPH oxidase of neutrophils is a multisubunit enzymatic complex responsible for the monoelectronic reduction of oxygen to produce superoxide anion (O_2^-^_) [9]. Free radical production is directly related to the bactericidal capacity of these cells since patients with chronic granulomatous disease (CGD), whose NADPH oxidase is inactive [10], suffer recurrent bacterial and fungal infections. The NADPH oxidase comprises the cytosolic factors p47^*phox*^, p67^*phox *^and p40^*phox*^, the membrane-associated cytochrome *b*_558 _and the accessory proteins Rac2 and Rap1a. The cytochrome *b*_558_, consisting of the glycoprotein gp91^*phox *^and the protein p22^*phox*^, localizes in the plasma membrane as well as in the membrane of secretory vesicles, specific and tertiary granules. In resting neutrophils, the oxidase remains unassembled and, therefore, inactive. In response to adequate stimuli, the cytosolic factor p47^*phox *^is phosphorylated at serine residues located at its carboxy terminus, then the phox homology (PX) domain located in its amino terminus is unmasked [11] and p47^*phox*^, together with p67^*phox*^, translocates to the membrane-associated cytochrome *b*_558_. This switches the NADPH oxidase to its active form [12]. The activation of the NADPH oxidase by a soluble stimulus like the formylated peptide N-formyl-L-methionyl-L-leucyl-L-phenylalanine (fMLP) involves both early trafficking of the cytochrome *b*_558 _to the plasma membrane through degranulation and the subsequent translocation of the cytosolic factors to assemble the oxidase. The exposure of neutrophils to phorbol 12-myristate 13-acetate (PMA) stimulates a larger degree of degranulation than that observed in response to fMLP and induces the assembly of the NADPH oxidase not only at the plasma membrane but also at the membrane of the intracellular vesicles/granules [13]. If neutrophils are exposed to particulate stimulus like opsonized microorganisms, phagocytosis takes place, and specific and azurophilic granules fuse with the phagosome to integrate the cytochrome *b*_558 _with the phagosomal membrane [10] and to release their contents into the phagolysosome [14]. In all cases, the activation of the NADPH oxidase requires the interaction of p47^*phox *^and p22^*phox*^, which is mediated by the SH3 domains located in the carboxy terminus of p47^*phox *^[15]. Recent studies suggested that the PX domain of p47^*phox*^, a phosphoinositide-binding module [16], plays an important role in the translocation of p47^*phox *^towards biological membranes [17]. We show that the PX domain of p47^*phox *^does not translocate to the membranes of mature phagosomes during phagocytosis of opsonized-zymosan particles.

## Results and discussion

### Transfection efficiency and cellular viability

**Figure 1:**
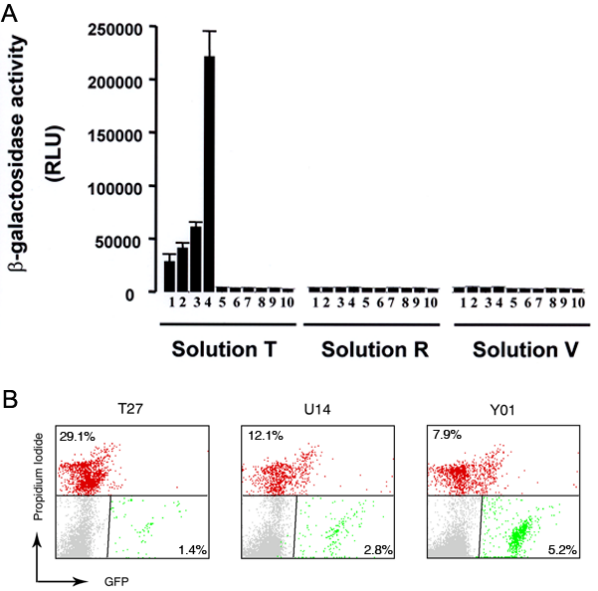
**Optimization of transfection and gene expression in human neutrophils**. A, O*ptimization of transfection and expression of the β-galactosidase gene in human neutrophils*. Neutrophils were resuspended in the indicated amaxa solution, in the presence of the pCMV-β-gal expression vector (1–9) or the empty vector pCMV-PA (10), and transfected as described in "Methods." The programs used were as follows: 1, "A23"; 2, "A27"; 3, "T20"; 4, "T27"; 5, "T16"; 6, "T01"; 7, "G16"; 8, "O17"; 9, no pulse and 10, "T16". Transfection of pCMV-PA resulted in no detectable activity for any of the programs listed. These data represent the mean and SE of six separate experiments. B, Neutrophils (2 × 10^6 ^cells) were resuspended in solution "T" and transfected with the expression vector pmaxGFP (amaxa biosystems) as described in "Methods" using the electrical settings corresponding to programs T27, U14 or Y01. After a 2-hour recovery period, dead cells were stained with propidium iodide and the samples were analyzed by flow cytometry.

**Figure 2:**
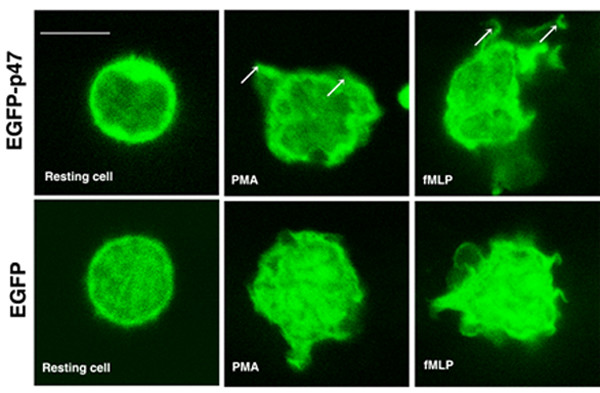
**Transfected neutrophils are responsive to soluble stimuli**. Neutrophils were transfected with the expression vector pEGFP-p47^*phox *^or with the pEGFP control vector as follows: Neutrophils were resuspended in solution "T" in the presence of the expression vector pEGFP and DNA was transfected using nucleofector program T27. The neutrophils were transferred to a poly-L-lysine – coated chambered glass slide containing 10% FCS-RPMI. Two hours after transfection, the cells were stimulated with PMA (0.1 μg/ml) or fMLP (1 μM) at 37°C for 15 min or 5 min, respectively. The cells were fixed with 3.7% paraformaldehyde and analyzed by confocal microscopy. Transfected neutrophils stimulated with PMA or fMLP can undergo morphological changes represented by the apparition of protrusions of the plasma membrane (arrows). Scale bar = 5 μm. These results are representative of four separate experiments.

To optimize the transfection of human neutrophils, we used the β-galactosidase expression vector pCMV-βgal, previously described [18]. After transfection, cells were incubated for 2, 4, 6 or 8 hours at 37°C and at the appropriate times were centrifuged, washed twice with PBS and lysed by sonication in 2% Nonidet P-40. β-Galactosidase activity was then measured using the β-gal Reporter Gene assay kit (Roche). Maximum galactosidase activity was observed at 2 h after gene transfer when using the proprietary transfection solution ''T'' (amaxa biosystems, Germany) and an electrical setting corresponding to program T27 (Fig. 1A). The transfection efficiency under these experimental conditions was 0.4 to 1%. The viability of neutrophils after the electrical pulse and after the 2-hour recovery period was 78.8 ± 2.5% and 78.5 ± 2.3%, respectively, evaluated using Trypan Blue exclusion. Next, we compared the efficiency of transfection of human neutrophils under various electrical settings corresponding to a series of second generation programs for amaxa nucleofectors. In figure 1B, we show that the electrical settings corresponding to programs U14 and Y01 resulted in higher transfection efficiency for similar experimental conditions based on flow cytometric analysis of neutrophils transfected with the vector pmaxGFP. Also in figure 1B, we show that the second generation programs (U14 and Y01) better preserve cell viability when evaluated by propidium iodide. For consistency and since the initial experiments were performed using program T27, this electrical setting was employed in subsequent experiments unless stated otherwise. Importantly, no differences in the subcellular distribution of the fluorescent chimeras were observed when using program Y01 instead of T27. Next, we used confocal microscopy to examine the transfection and expression of enhanced green fluorescent proteins encoded by the pEGFP (EGFP, enhanced green fluorescent protein) expression vector (Clontech^®^) in neutrophils. To demonstrate further that the population of transfected neutrophils was fully functional, we transfected neutrophils with the NADPH oxidase cytosolic factor p47^*phox *^as an EGFP chimera, and we stimulated cells with phorbol ester or with the formylated peptide fMLP which mimics bacteria-derived peptides and stimulates neutrophils through binding to the membrane receptor formyl peptide receptor 1 (FPR1) [19]. In figure 2, we show that transfected neutrophils stimulated with PMA or fMLP can undergo morphological changes represented by the appearance of protrusions of the plasma membrane, presumably membrane ruffles. These membrane structures were enriched in EGFP-p47^*phox *^(Fig. 2), which is in agreement with previous reports describing the localization of NADPH oxidase factors at membrane ruffles after activation in non-primary cell lines [20]. The distribution pattern for EGFP was different from that of EGFP-p47^*phox*^. EGFP was detected in the nucleus and cytosol, and was also detected in membrane protrusions in stimulated cells (Fig. 2).

**Figure 3:**
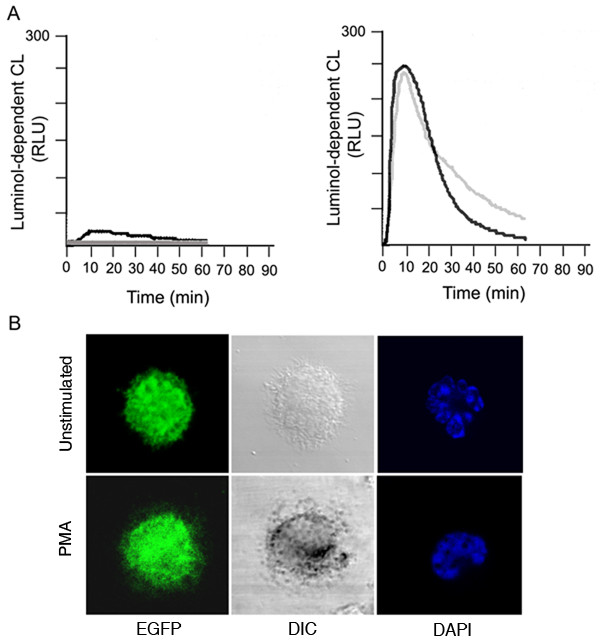
**Nucleoporated neutrophils conserve a responsive NADPH oxidase**. A, Neutrophils were resuspended in solution "T" and transfected using an EGFP expression vector and an electrical setting corresponding to nucleofector program T27 (black lines) or incubated with DNA without electrical pulse (grey lines). The cells were recovered in serum-free RPMI containing 0.1% gelatin. Neutrophils (2 × 10^6^) were left untreated (left panel) or stimulated with PMA (right panel). Luminol-dependent chemiluminescence was continuously recorded for 65 min. The results are representative of three different experiments. B, Neutrophils were nucleoporated with an expression vector coding for EGFP, recovered for 2 h in RPMI and stimulated with PMA in the presence of Nitroblue Tetrazolium for 30 min at 37°C. After stimulation, cells were washed with PBS and fixed. Nuclei were visualized using the DNA-labeling fluorescent compound 4',6-Diamidino-2-phenylindole (DAPI). The samples were then analyzed by confocal microscopy and by differential interference contrast (DIC) microscopy.

**Figure 4:**
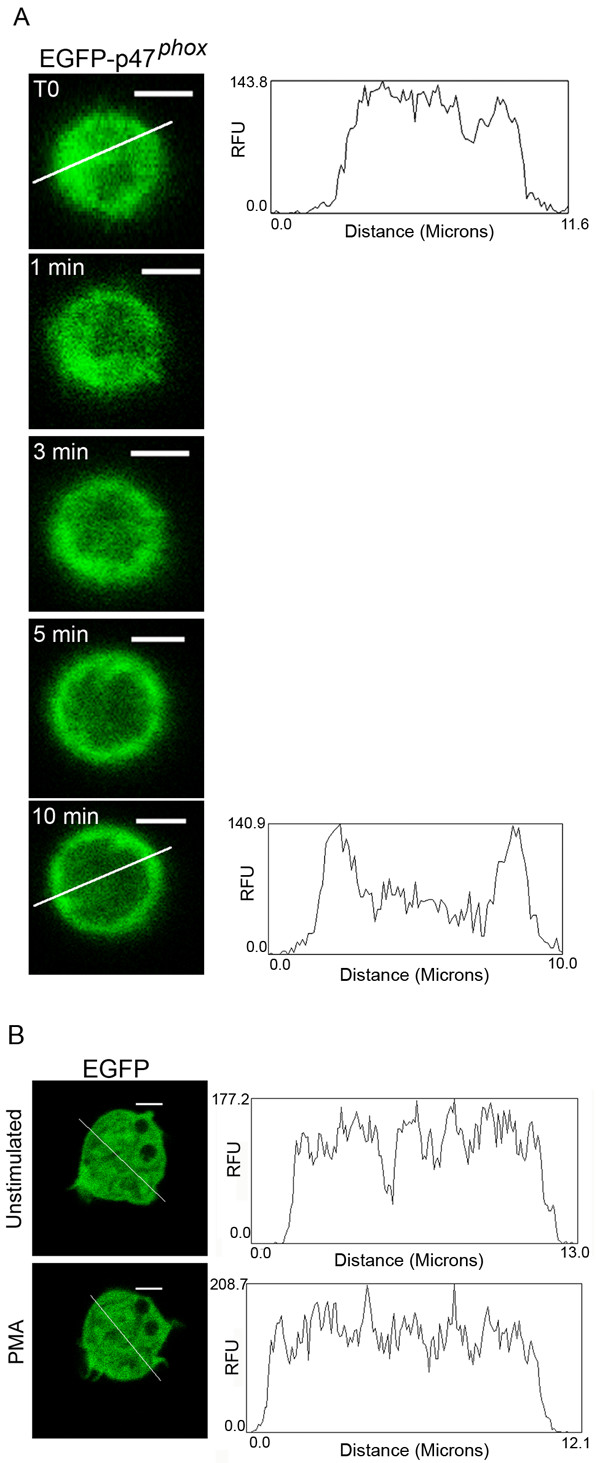
**EGFP-p47^*phox *^translocates to the plasma membrane in nucleoporated neutrophils**. Neutrophils were nucleoporated with a vector expressing EGFP-p47^*phox *^or EGFP control vector and recovered in RPMI. Two hours after transfection the specimens were transferred to a live chamber (37°C and 4.9% CO_2_) and cells were stimulated with PMA (0.1 μg/ml). The distribution of the fluorescent chimera was analyzed by confocal microscopy at the indicated intervals. The images were processed and analyzed for the distribution of the fluorescence intensity using the NIH image processing and analysis program IMAGE/J software. Scale bars = 3 μm.

To evaluate the effect of nucleofection on NADPH oxidase activation, we first analyzed the ability of nucleoporated neutrophils to produce ROS using the luminol-dependent chemiluminescence detection system. In figure 3A, we show that electroporated cells have relatively low basal luminol-dependent chemiluminescence activity in the absence of stimuli. Importantly, they maintain their capacity to respond to stimuli. Nucleoporated neutrophils show ROS production kinetics similar to that of non-electroporated cells in response to PMA (Fig. 3A). Furthermore, electroporated neutrophils expressing an exogenous protein respond to stimulus (Fig. 3B). The response was detected using the nitroblue tetrazolium reaction. Formazan, the blue-black precipitate generated by superoxide anion-dependent NBT reduction, was evident in stimulated cells but undetectable in unstimulated, transfected cells (Fig. 3B). These results suggest that electroporated neutrophils conserve an intact NADPH oxidase. To further support this idea, we transfected neutrophils with a vector expressing wild type p47^*phox *^downstream of EGFP and then stimulated the cells with PMA. The translocation of p47^*phox *^to the plasma membrane was followed in real time by confocal microscopy analysis (Fig. 4). The results indicate that EGFP-p47^*phox*^, but not the EGFP control, undergoes translocation during activation further supporting the idea that electroporated neutrophils are functional and responsive.

**Figure 5:**
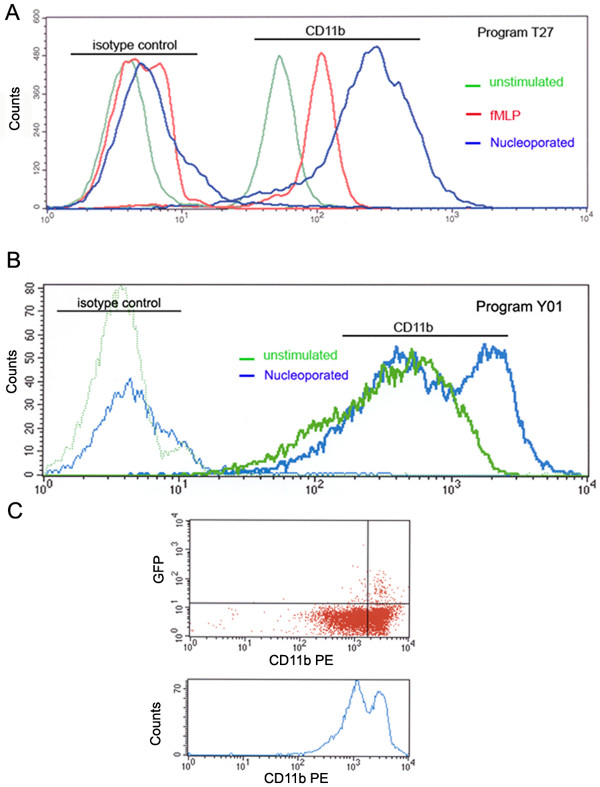
**Surface expression of CD11b in nucleoporated neutrophils**. The expression of the human leukocyte integrin subunit CD11b on the surface of nucleoporated neutrophils was analyzed by flow cytometry as described in "Methods." A, Cells were transfected using solution "T" and an electrical current corresponding to program T27. Nucleoporated cells were recovered for 2 h in serum-free RPMI medium (blue lines). Untransfected cells (no pulse) were recovered in RPMI and, where indicated, stimulated with fMLP for 5 min (red lines) or left untreated (green lines). The cells were labeled with anti-CD11b or an isotype-matched control. Expression of CD11b antigen on the surface of treated and untreated neutrophils was analyzed by flow cytometry. The results are representative of three independent experiments. B, Cells were transfected using solution "T" and an electrical current corresponding to program Y01 (blue lines) or not pulsed (green line). The cells were labeled for surface expression of CD11b as described above. The results are representative of three independent experiments. C, Cells were transfected with the expression vector pmaxGFP using the electrical setting corresponding to program Y01. After a 2 h recovery period, the cells were analyzed for the detection of the surface expression of CD11b using a specific antibody conjugated to phycoerythrin (PE, BD Biosciences). The samples were analyzed by flow cytometry as described under "Methods."

To evaluate whether nucleofection induces more subtle degrees of activation in neutrophils, we examined the surface expression of CD11b, a human leukocyte integrin subunit that is mainly present in the membrane of secretory vesicles and tertiary granules in resting neutrophils. In figure 5, we show that nucleoporated neutrophils have, in fact, a marked increase in CD11b molecules on their cellular surface. The level of CD11b at the plasma membrane after nucleoporation was even larger than that triggered by fMLP stimulation. The increase in the surface expression of CD11b was evident using either program T27 or the second generation program Y01 (Fig. 5A and 5B). In some experiments, two populations of cells were identified according to their level of surface expression of CD11b after nucleoporation (Fig. 5B). Cells expressing GFP were mainly distributed with the subpopulation of cells showing higher surface expression of CD11b (Fig. 5C). These results suggest that nucleofection induces the mobilization of secretory vesicles and, probably, tertiary granules, which are the subpopulation of neutrophil secretory organelles with the highest tendency to undergo exocytosis.

### The PX domain of p47^*phox *^does not translocate to the phagosomal membrane in human neutrophils

**Figure 6:**
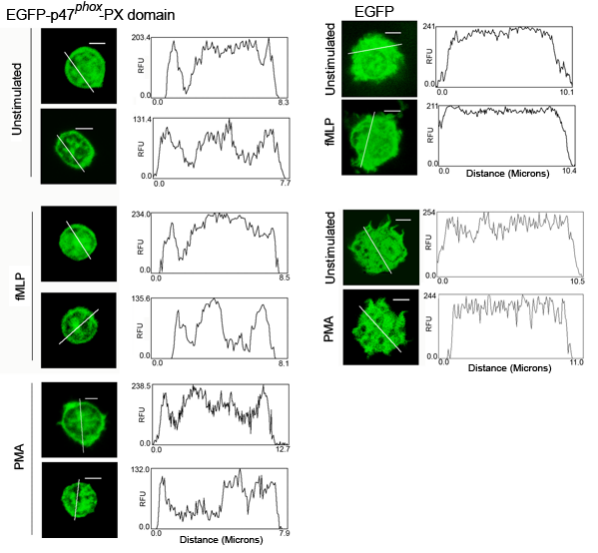
**The PX domain of p47^*phox *^localizes at the plasma membrane in resting and stimulated neutrophils**. Neutrophils were nucleoporated with the expression vector EGFP-p47-PX or with the EGFP control vector. After 2 h, the cells were stimulated with fMLP (1 μM), PMA (0.1 μg/ml) or left untreated. The cells were then fixed and analyzed for fluorescence distribution by confocal microscopy. The images were processed and analyzed for the distribution of the fluorescent proteins using the NIH image processing and analysis program IMAGE/J software. Scale bars = 3μm. For EGFP-p47-PX, separate cells are shown. For EGFP, the same cells are shown before and after stimulation. The results are representative of three independent experiments.

The cytosolic factors p47^*phox *^and p40^*phox *^each contain a PX domain at their amino terminus [16]. PX domains are also present in proteins involved in vesicular trafficking and cellular signaling including Vam7p [21] and PI3K-C2 [22,23], respectively. PX domains are phosphoinositide binding modules [16]. The affinity of the various PX domains for several phosphoinositides differs from protein to protein. In particular, the PX domain of p47^*phox *^has been shown to bind preferentially to PI(4)P, PI(3,4)P_2 _[16], and PI(3,4,5)P_3 _[20]. The mechanism mediated by the PX domain of p47^*phox *^in the activation of the oxidase is controversial. One group has suggested that the phosphoinositide-binding activity of the p47^*phox *^PX domain is essential for the membrane translocation of this protein and activation of the phagocyte NADPH oxidase [11]. Other groups have indicated that the translocation of the p47^*phox *^PX domain to the plasma membrane is not due to interactions with phospholipids but to association with the actin cytoskeleton [24] and have shown in a variety of cell lines that the PX domain of p47^*phox *^targets to cell membranes via interaction with moesin [24,25]. Furthermore, while some researchers propose that a single residue substitution in the PX domain of p47^*phox *^decreases its affinity to phosphoinositides and its binding to cell membranes in resting or PMA-stimulated cells [11], others show that the PX domain of p47^*phox *^translocates to the plasma membrane after activation in a phosphoinositide-independent manner in COS cells [24]. The subcellular localization of the PX domain of p47^*phox *^in human neutrophils has not yet been shown. To analyze this, we transfected human neutrophils with a vector for the expression of a chimera composed of EGFP upstream of the p47^*phox *^PX-domain (EGFP-p47-PX, Fig. 6). The visualization of the subcellular distribution of the PX domain is somewhat difficult due to the relatively small size of these cells. However, the fluorescence intensity plots helped to identify an increase in the distribution of fluorescence towards the edge of the cells. We concluded that the p47^*phox *^PX domain partially localizes at the plasma membrane in nucleoporated neutrophils. Localization of EGFP-p47-PX in intracellular structures was also evident, a phenomenon previously described in COS cells [20]. Differently from the fusion protein EGFP-p47^*phox*^(full-length) which required phorbol ester-mediated neutrophil activation for plasma membrane localization (Fig. 4), the subcellular distribution of EGFP-p47-PX was not markedly affected by cellular stimulation (Fig. 6). Although the concentrations of PI(3,4)P_2 _[16], and PI(3,4,5)P_3 _in resting neutrophils are considered to be relatively low [26], it could be possible that PI3-kinases undergo some level of activation during nucleoporation thus increasing the membrane distribution of the PX domain in unstimulated cells. However, pretreatment of neutrophils with the PI3-kinase inhibitor LY294002 did not alter the pattern of protein distribution in EGFP-p47-PX transfected neutrophils or prevent the increase in the surface expression of CD11b after nucleoporation (not shown). Another possibility is that once exposed (the PX domain is masked in unphosphorylated full-length p47^*phox*^), the PX domain could recognize pre-existent structures at the plasma membrane, possibly moesin, PI(4)P [16], phosphatidic acid [27] or even basal levels of PI 3-kinase products. From our experiments, it seems that the plasma membrane of nucleoporated neutrophils has the necessary molecular machinery to sequester, at least in part, the p47^*phox *^PX-domain. The molecular basis of this mechanism remains to be clarified.

**Figure 7:**
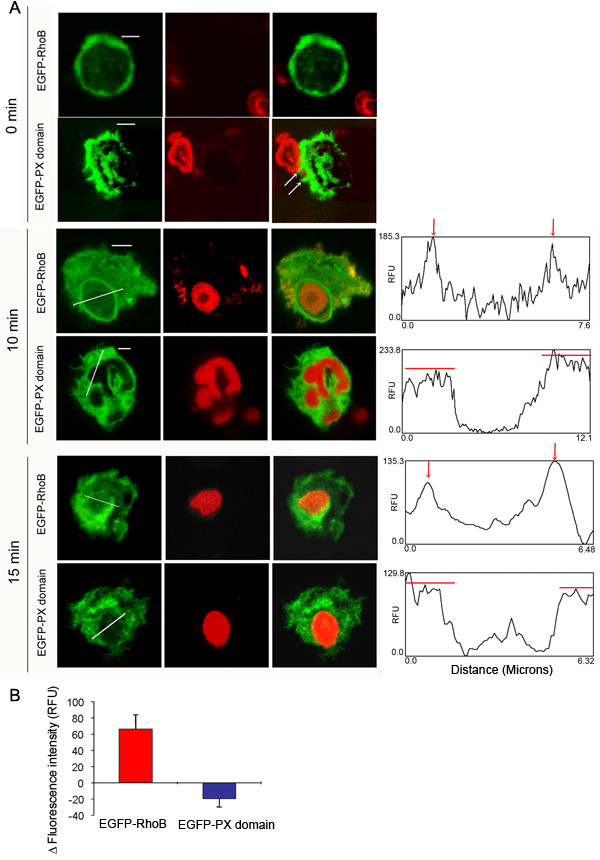
**Distribution of exogenously expressed p47^*phox*^-PX domain and RhoB during phagocytosis in human neutrophils**. Neutrophils were nucleoporated with the expression vectors EGFP-p47-PX or EGFP-RhoB as described in figure 2. After a 2 h recovery period, cells were incubated in the presence of Texas Red-labeled opsonized-zymosan particles at 37°C for 10 or 15 min. Samples were washed, fixed and analyzed by confocal microscopy as described in "Methods." The images were processed and analyzed for the distribution of the fluorescent proteins using the NIH image processing and analysis program IMAGE/J software. The plot profiles, in the right panel, show that EGFP-RhoB is accumulated around the phagosome (red arrows indicating peak of fluorescence intensity) while the plot profiles corresponding to EGFP-p47-PX show a plateau of fluorescence intensity (red lines). The white arrows show the distribution of p47^*phox*^-PX domain in membrane protrusions surrounding the opsonized particle at time zero. Scale bar = 3 μm. B, The difference between the fluorescence intensity at the phagosome membrane versus that at the surrounding cytosolic area (ΔF_I_) was calculated using the NIH image processing and analysis program IMAGE/J software as described under "Methods." The results are mean ± SEM of three phagosomes from different cells.

**Figure 8:**
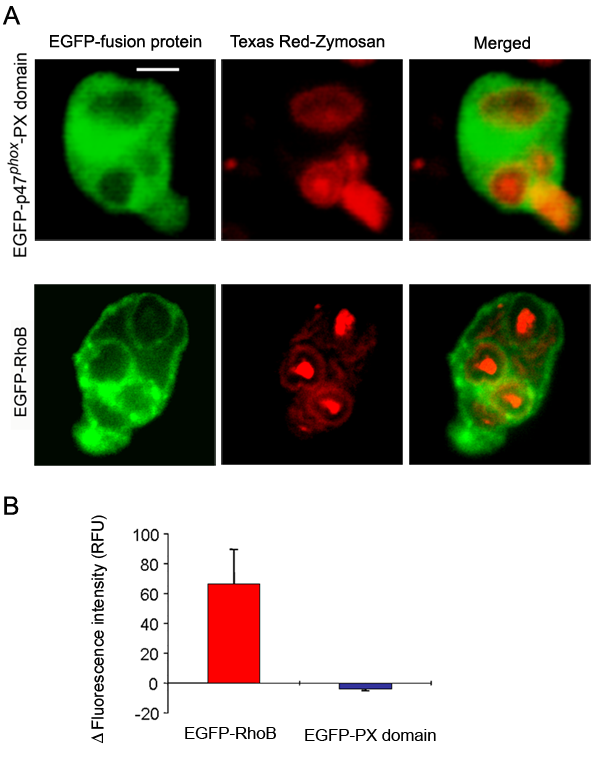
**Distribution of exogenously expressed p47^*phox*^-PX domain and RhoB during phagocytosis in HL-60 human promyelocytic cells **HL-60 promyelocytic cells were differentiated to granulocytes and transfected with the indicated expression vector as described under "Methods." Phagocytosis of Texas Red-labeled opsonized-zymosan particles was conducted at 37°C for 15 min. B, The difference between the fluorescence intensity at the phagosome membrane versus that at the surrounding cytosolic area (ΔF_I_) was calculated using the NIH image processing and analysis program IMAGE/J software as described under "Methods." The results are mean ± SEM of three phagosomes from different cells.

During particle engulfment, the NADPH oxidase complex is assembled at the membrane of the phagosome so that superoxide anion is pumped into the core of the phagosome where the engulfed microorganism is killed. In particular, p47^*phox *^has been shown to localize towards nascent and mature phagosomes in neutrophils after phagocytosis of opsonized zymosan particles [10]. This poses the question of whether the PX domain of p47^*phox*^, which has been proposed to mediate the translocation of p47^*phox *^towards the cytochrome *b*_558_-containing plasma membrane and to participate in the activation of the NADPH oxidase in cells stimulated with soluble stimuli [11], could also be implicated in the assembly of the oxidase at the phagosome membrane. In an attempt to clarify this, we analyzed the distribution of the PX domain of p47^*phox *^during phagocytosis. We transfected neutrophils with the expression vector pEGFP-p47-PX or with cDNA encoding a chimera of EGFP and RhoB, a small GTPase thought to function in the regulation of endocytosis [28] and phagocytosis [29]. Then, we exposed the transfected cells to Texas Red-conjugated opsonized zymosan A particles, and protein distribution during phagocytosis was evaluated by confocal microscopy. In Figure 7, we show that neutrophils expressing EGFP-RhoB or EGFP-p47-PX can undergo phagocytosis. From those experiments, it becomes clear that nucleoporated neutrophils conserve their ability to phagocytose opsonized particles. The accumulation of green fluorescence around the phagosome is evident in EGFP-RhoB-expressing neutrophils but was not observed in cells expressing EGFP-p47-PX (Fig. 7). Similar results were observed in HL-60 promyelocytic cells when differentiated to granulocytes (Fig. 8). These data suggest that the mechanisms of translocation of p47^*phox *^to the plasma membrane and to the phagosome membrane are different. It is likely that the translocation of p47^*phox *^to the phagosome does not involve the PX domain. One possibility is that the SH3 domains of p47^*phox*^, which have been largely shown to bind to the membrane-localized cytochrome *b*_558 _through interaction with the cytosolic domain of p22^*phox*^, are sufficient to translocate p47^*phox *^to the phagosome. However, p47^*phox *^was clearly detected in forming phagosomes in X-linked CGD neutrophils which lack cytochrome *b*_558_, indicating that the recruitment of this protein to the nascent phagosome is independent of cytochrome *b*_558 _[10]. In the same work, p47^*phox *^was undetectable in phagosome membranes of mature phagosomes in neutrophils from X-linked CGD patients [10], suggesting that p47^*phox *^can not be retained at the phagosome in the absence of cytochrome *b*_558_. Likewise, the PX domain of p47^*phox *^localizes at the plasma membrane in the proximity of the opsonized particle in the nascent phagosome (Fig. 7), but is not retained in the membrane of mature phagosomes (Fig. 7 and 8) despite the fact that moesin, the actin-binding protein shown to bind to the PX domain of p47^*phox*^, is present in phagosome membranes [30]. Therefore, the reason why the PX domain of p47^*phox *^is not retained in the mature phagosome may reside in the differential role that the products of class I and class III phosphatidylinositol 3-kinase play during phagosome formation and maturation [31]. The product of class I PI 3-kinase, PI(3,4,5)P3, has been shown to be rapidly synthesized during phagosomal formation [32,33], but the accumulation was sharply restricted to the phagosomal cup [32]. Conversely, PI(3)P, a product of class III PI 3-kinase, was only detected in sealed phagosomes [31]. These data correlate with our observation that the PI(3,4,5)P3-binding domain of p47^*phox *^(PX domain) is present in membranes in the forming phagosome but is absent from the membrane of the mature phagosome. Although not explored here, it is also possible that the PI(3)P-binding domain of p40^*phox *^(p40^*phox*^-PX domain) is implicated in maintaining the cytosolic complex of the NADPH oxidase at the mature phagosome.

## Conclusion

We demonstrated that cDNA transfer and expression of exogenous protein in human neutrophils is compatible with cell viability and is no longer a limitation on the study of neutrophil function; however, the relatively low efficiency of transfection/expression observed after 2 h restricts the subsequent analysis to experiments that use single cells. Since phagocytosis of opsonized particles by neutrophils as well as their response to stimuli requires intact downstream signaling machinery, the results presented here suggest that transfected neutrophils are viable and fully functional. Using this methodology, we showed that the PX domain of p47^*phox *^is translocated to the plasma membrane but is not retained at the membrane of mature phagosomes suggesting that distinct mechanisms may operate during the activation of the NADPH oxidase at different subcellular sites in human neutrophils.

## Methods

### Transfection of human neutrophils and confocal microscopy

For our studies, we isolated neutrophils from healthy human donors as previously described [34] and resuspended them in phosphate-buffered saline (PBS). Neutrophils were then stored on ice for 30 min or less before use. Immediately before transfection, cells were centrifuged at 1,800 rpm (800 × g) and 4°C for 5 min then resuspended in transfection buffer as indicated below. Ninety μL of neutrophil suspension (2 × 10^6 ^cells) were transferred to a nucleoporation cuvette (amaxa Biosystems, Germany), and 5 μg of the indicated cDNA were added to complete a final volume of 100 μl. We found that this concentration of cells is essential to achieve maximum transfection efficiency. Cells were transfected in an amaxa nucleofector apparatus then immediately transferred to 8-well poly-L-lysine–coated chambered glass slides (Lab-Tek) containing RPMI medium supplemented with 10% fetal calf serum (50,000 to 100,000 cells per well in 400 μl of medium). Neutrophils were maintained for 2 h at 37°C in 5% CO_2_/air then fixed with 3.7% paraformaldehyde for 10 min. Fixed cells were washed three times with PBS and stored in Fluoromount-G (Southern Biotechnology, CA) until analysis by laser-scanning confocal microscopy on either a Bio-Rad MRC1024 attached to a Zeiβ Axiovert S100TV microscope or a *Zeiss (BioRad) *Radiance 2100 Rainbow laser scanning confocal microscope (LSCM) attached to a Nikon TE2000-U microscope with infinity corrected optics. Images were collected using the Bio-Rad LaserSharp (v3.2) software. Images were taken at constant exposure times pre-determined to be sub-saturating for the brightest sample. The images were processed and analyzed for the distribution of the fluorescence intensity using the NIH image processing and analysis program IMAGE/J software, IMARIS software (Bitplane AG) and Image-pro plus (MediaCybrnetics^®^). In some experiments, neutrophils were stimulated 2 h after transfection using the formylated peptide fMLP (1 μM) or PMA (0.1 μg/ml) for 5 or 20 min, respectively, at 37°C. Where indicated, the subcellular localization of the EGFP-p47^*phox *^chimera was followed in real time using a *Zeiss *Radiance 2100 Rainbow LSCM attached to a Nikon TE2000-U microscope equipped for viewing live specimens with a Neue temperature and CO_2 _controlled live chamber (LiveCell Inc., PA) and a Bioptechs Objective heater (Biotechs, Inc., PA).

### Phagocytosis assay

Neutrophils were transfected as described above with the expression vectors EGFP-RhoB or EGFP-p47^*phox*^-PX. After transfections, the cells were maintained for 2 h at 37°C in RPMI medium at a cellular concentration of 100,000 neutrophils per 400μl of RPMI in 8-well poly-L-lysine – coated chambered glass slides (Lab-Tek). Then, the cells were incubated in the presence of Texas Red-conjugated zymosan A (*S. cerevisiae*) BioParticles (Invitrogen, CA), previously opsonized using the Fluorescent Particles Opsonizing Reagent (Invitrogen, CA) as described by the manufacturer. The fluorescent particles were added in a ratio of particles to phagocytes 15:1 or 100:1, the slides were immediately spun down at 1,500 rpm for 5 min at 4°C then incubated for 10 or 15 min at 37°C. Cells were fixed, washed with PBS and stored in Fluoromount-G (Southern Biotechnology) at 4°C until analyzed by laser-scanning confocal microscopy as described above. The quantification of the fluorescent intensity (F_I_) at the phagosomal membrane versus the cytosolic distribution of the fluorescent chimera for the green channel was assessed using the NIH image processing and analysis program IMAGE/J software. Briefly, two lines were drawn on each phagosome using the straight line tool. These generated four points where the lines intersected the phagosomal membrane. The intensity of fluorescence at the end of the lines (~ 1 μm outside the phagosome into the cytosol) was subtracted from the fluorescence intensity at the point where the lines intersected the phagosome (ΔF_I_). The four independent values were averaged and used as representative of the ΔF_I _for that particular phagosome. At least three phagosomes from different cells were analyzed for each chimera.

### Nitroblue tetrazolium test

Human neutrophils were nucleoporated as described above and maintained in RPMI medium for 2 h at 37°C in 8-well chambered coverglass slides at 50.000 cells/well). Then, cells were incubated with 1 mg/ml Nitroblue tetrazolium (NBT) (Bio-Rad Laboratories, CA) in the presence or absence of 0.1 μg/ml PMA, for 30 min at 37°C. After stimulation, cells were washed with PBS, fixed and analyzed by confocal microscopy.

### Luminol-dependent chemiluminescence assay

Neutrophils were nucleoporated in solution "T" using an EGFP expression vector and an electrical setting corresponding to program T27. The cells were recovered in serum-free RPMI containing 0.1% gelatin (Sigma, MO) for 2 h at 37°C. Neutrophils (2 × 10^6^) were resuspended in PBS containing 5 mM glucose and 0.1% gelatin. Luminol was added to a final concentration of 1 μM. Cells were left untreated or stimulated with 0.1 μg/ml PMA. Luminol-dependent chemiluminescence was continuously recorded for 65 minutes.

### Flow cytometry analysis

Neutrophils were nucleoporated in solution "T" using an electrical setting corresponding to programs T27, U14 or Y01 in the presence or absence of the expression vector pmaxGFP (amaxa biosystems). The cells were recovered in serum-free RPMI containing 0.1% gelatin (Sigma, MO) for 2 h at 37°C. Untransfected cells were also incubated in RPMI and used as a control. Where indicated, control cells were stimulated with fMLP (1 μM). The cells were spun down, washed and resuspended in flow cytometer diluent buffer (PBS containing 0.5% BSA and 3 mM NaN_3_). Cells were incubated with a specific antibody anti-CD11b or with an isotype-matched control (BD Pharmingen, CA). Next, they were incubated with a fluorescein isothiocyanate-conjugated anti-mouse antibody (Jackson ImmunoResearch Laboratories, PA) then fixed in 1% paraformaldehyde. Expression of CD11b antigen on the surface of treated and untreated neutrophils was analyzed by flow cytometry (FACSCalibur BD Biosciences, CA). The data was analyzed using CellQuest™ software (Becton Dickinson, CA). For flow cytometry based viability analysis, cells were labeled for 15 min with propidium iodide (final conc. 10 μg/ml) and analyzed by flow cytometry. GFP fluorescence was detected in the FL-1 channel and propidium iodide using the FL-3 channel.

### Plasmid construction

The various steps in the cloning of the constructs used in this work were performed by standard techniques, and all constructs were verified by sequencing using an automated fluorescent dye-terminator sequencer. The full-length p47^*phox *^and the PX domain of p47^*phox *^(residues 1–130) cDNA were amplified from the full-length cDNA using *pfu *polymerase (Stratagene, La Jolla, CA) and the following primers: 5' primer GAATTCATGGGGGACACCTTCATCCGT; p47*^phox^* 3' primer GGTACCGACGGCAGACGCCAGCTTCCG and PX domain 3' primer, GGTACCGTCTGTGGGGAGCTTGAGGT. The *Eco*RI I and *Kpn*I sites are underlined. The fragments were purified and ligated into the pEGFP-C2 vector (Clontech).

### Transfection of HL-60 promyelocytic cells

The promyelocytic leukemia human HL-60 cell line (American Type Culture Collection (ATCC), VA) was cultured in Dulbecco's Modified Eagle Medium (D-MEM) (Gibco) supplemented with 20% fetal bovine serum (Hyclone), 0.292 mg/ml glutamine, 50 units/ml penicillin and 50 μg/ml streptomycin at 37°C in 5% CO_2_/air. HL-60 cells were differentiated to granulocytes by incubation in the presence of 1.3% DMSO for 48 h. For transfections, 5 × 10^6 ^cells were resuspended in 100 μl of Solution "V" (amaxa biosystems) in the presence of 5 μg of the vectors expressing EGFP-p47-PX domain or EGFP-RhoB and nucleofected in the nucleoporator apparatus (amaxa Biosystems, Germany) using the T01 electrical setting. The cells were then re-plated in complete medium in the presence of 1.3% DMSO, incubated at 37°C in 5% CO_2_/air and used for analysis 24 h post-transfection. For phagocytosis assays, transfected HL-60 cells were seeded at 70% confluence in an eight-well chambered coverglass (pre-treated with 0.01% poly-L-lysine in PBS) in D-MEM medium for 30 min at 37°C and incubated in the presence of Texas Red^®^-conjugated zymosan A (*S. cerevisiae*) BioParticles^® ^(Invitrogen), that had been opsonized using the Fluorescent Particles Opsonizing Reagent (Invitrogen) as described by the manufacturer. The fluorescent particles were added in a ratio of particles to phagocytes approximately 15:1 and the slides were immediately spun down at 1,500 rpm for 5 min at 4°C then incubated for 15 min at 37°C. Next, cells were fixed with 3.7% PAF, washed with PBS, and stored in Fluoromount-G (Southern Biotechnology) at 4°C until analysis by laser-scanning confocal microscopy

### Control of protein expression

HL-60 granulocytes were transfected with the expression vectors pEGFP, pEGFP-p47^*phox *^or pEGFP-p47^*phox*^-PX as described above. The cells were lysed using M-PER mammalian protein extraction reagent (Pierce) in the presence of anti-proteases and 40 μg of total protein was resolved by SDS-PAGE, transferred to nitrocellulose and detected using a monoclonal antibody raised against the GFP tag (B-2, Santa Cruz Biotechnology).

### Human subjects

All procedures regarding human subjects have been reviewed and approved by the Human Subjects Research Committee at The Scripps Research Institute and were conducted in accordance with the requirements set forth by the mentioned Human Subjects Research Committee.

## Authors' contributions

JLJ contributed to the experimental design, set up conditions for the transfection experiments, optimized the transfection and expression of the β-galactosidase gene in human neutrophils, performed some of the phagocytosis experiments and contributed to the writing of the manuscript. BAE isolated human neutrophils, contributed to the optimization of the phagocytosis assays and performed flow cytometry assays. DBM contributed to the analysis of the confocal microscope images, performed Western blots, phagocytosis assays and NBT tests. AAB designed and performed flow cytometry assays and analyzed results. SDC designed nucleofection experiments for transfection of human neutrophils, performed research, collected and processed confocal microscopy images and wrote the manuscript. All of the authors read and approved the final manuscript.
